# Targeting LKB1 in cancer – exposing and exploiting vulnerabilities

**DOI:** 10.1038/bjc.2015.261

**Published:** 2015-07-21

**Authors:** M Momcilovic, D B Shackelford

**Affiliations:** 1 Department of Pulmonary and Critical Care Medicine, David Geffen School of Medicine, University of California, Los Angeles, CA 90095, USA

**Keywords:** LKB1, cancer, therapeutic strategies, vulnerabilities

## Abstract

The LKB1 tumour suppressor is a serine/threonine kinase that functions as master regulator of cell growth, metabolism, survival and polarity. LKB1 is frequently mutated in human cancers and research spanning the last two decades have begun decoding the cellular pathways deregulated following LKB1 inactivation. This work has led to the identification of vulnerabilities present in LKB1-deficient tumour cells. Pre-clinical studies have now identified therapeutic strategies targeting this subset of tumours that promise to benefit this large patient population harbouring LKB1 mutations. Here, we review the current efforts that are underway to translate pre-clinical discovery of therapeutic strategies targeting LKB1 mutant cancers into clinical practice.

Liver kinase B1 (LKB1, also known as STK11) was first identified as the causal mutation in Peutz–Jeghers Syndrome (PJS), a rare inherited autosomal dominant disorder characterised by the development of benign gastrointestinal hamartomas and the early onset of cancer (Hemminki *et al*, 1998). However, more than a decade later, LKB1 has become recognised as a critical tumour-suppressor gene that is frequently mutated in a broad spectrum of human cancers. LKB1 is a serine threonine kinase that directly phosphorylates and regulates the adenosine monophosphate-activated protein kinase (AMPK) and 12 other AMPK-like kinases to regulate a broad spectrum of cellular functions including: growth, metabolism, autophagy and polarity (Figure 1) (Shackelford and Shaw, 2009). The past decade has witnessed an intense effort to understand the molecular mechanisms that underlie basic cellular deregulation that occurs following LKB1 inactivation in cancer. Unlike targeting gain-of-function mutations with targeted inhibitors such as the drug Imatinib that inhibits the BCR-ABL tyrosine kinase driver mutation in chronic myeloid leukaemia (Druker *et al*, 2001), loss-of-function mutations in tumour suppressors present a unique therapeutic challenge. Owing to the lack of a clear target, a synthetic lethal approach is required to identify liabilities in growth and survival pathways in order to expose weak points in LKB1 mutant tumours that may be therapeutically exploited. Additionally, a clear understanding of the deregulation of key pathways downstream of LKB1 and its substrates will likely hold the key to developing effective therapies for the treatment of LKB1 mutant cancers. Here, we review the current strategies and research efforts to develop personalised treatments targeting tumours bearing LKB1 mutations.

## Stratifying Patients by LKB1 Loss of Function

The predisposition of PJS patients to early-onset tumours predicted that LKB1 inactivation is an important event in carcinogenesis (Hemminki, 1999). Subsequent genetic analysis of tumours has demonstrated that LKB1 is frequently inactivated in human tumours of the cervix, ovaries, skin, pancreas and, recently, the kidneys (Sanchez-Cespedes, 2007). LKB1-inactivating mutations are common in non-small cell lung cancer patients (NSCLC) through loss of heterozygosity on chromosome 19p that frequently accompanies nonsense and missense mutations as well as deletions (Fernandez *et al*, 2004). Non-small cell lung cancer patients is a heterogeneous disease comprising tumours of varying histopathological subtypes. LKB1 mutations were detected in approximately 20–30% of lung adenocarcinoma (ADC), 70% of mucinous bronchiolar ADC and to a lesser extent in squamous and large cell carcinomas (Sanchez-Cespedes *et al*, 2002; Ji *et al*, 2007; Osoegawa *et al*, 2011; Wilkerson *et al*, 2012). Moreover, The Cancer Genome Atlas (TCGA) project confirmed these studies and identified LKB1 as the third most frequently mutated gene in human lung ADC (Ding *et al*, 2008). Therefore, it reasons that in order to successfully treat LKB1 mutant cancers, patient biopsies must first be routinely screened for LKB1 inactivation.

It will be critical to implement standardised genetic and molecular screening of LKB1 inactivation for patients. However, routine screening for LKB1 gene mutations is not standard-of-care practice making it difficult to stratify patients by LKB1 inactivation. Accurate detection of LKB1 mutations require a combination of deep sequencing techniques including Sanger sequencing to detect point mutations and loss of heterozygosity as well as exon sequencing by Multiplex ligation-dependent probe amplification (Ji *et al*, 2007; Wingo *et al*, 2009). Immunohistochemical staining of total LKB1 protein on formalin-fixed paraffin-embedded biopsies may provide an effective alternative approach to costly sequencing as a means to identify the loss of LKB1 in cancer. Tumours were stratified by LKB1 loss in two biomarker studies examining LKB1 expression in NSCLC and head and neck squamous cell carcinoma (Kline *et al*, 2011; Nakada *et al*, 2013). However, immunohistochemical staining of total LKB1 protein does not distinguish between functional and non-functional LKB1. Additional staining of lung tumour biopsies for phosphorylated AMPK at threonine 172 (P-AMPKthr172) did show a significant and positive correlation between low LKB1 expression and low P-AMPKthr172 levels in lung tumours demonstrating the utility of using both LKB1 and P-AMPKthr172 as clinical biomarkers (Nakada *et al*, 2013). Examination of transcript levels of LKB1 may prove to be a more feasible and cost-effective method compared with multiple deep sequencing techniques. A recent study examining gene expression profiles in NSCLC identified a unique profile for LKB1 mutant lung tumours as compared with tumours expressing functional LKB1. Interestingly, the authors identified a large population of lung tumours with no detectable genetic mutations in LKB1 but exhibiting a LKB1 mutant gene expression profile. Examination of LKB1 transcript levels showed that these genetically wild-type but functionally mutant tumours had reduced LKB1 transcript levels confirming that the inactivation of LKB1 may occur at the genetic and transcriptional level (Kaufman *et al*, 2014). Additionally, in melanoma cells with BRAF V600E mutation, LKB1 can be inactivated by phosphorylation by ERK and Rsk leading to reduced AMPK phosphorylation levels (Zheng *et al*, 2009). The combination of deep sequencing, transcript and protein analysis combined would ensure the detection of LKB1 inactivation in tumours, however, this may not be the most practical route for clinical application. Therefore, functional assays that directly measure the LKB1 activation following treatment with AMPK activators such as AICAR or phenformin could in theory be performed on tumour biopsies cultured *ex vivo* (Shaw *et al*, 2004b; Hawley *et al*, 2010). This type of assay would require culturing and/or preserving tumour biopsies *ex vivo* and could serve as a simple and routine screening method to detect LKB1 deficiencies. Thus, multiplexing various assays is likely to prove the most effective approach to identify LKB1 inactivation in a broad spectrum of human tumours.

## Targeting Vulnerabilities in LKB1-Deficient Tumours

### Exploiting energetic stress

LKB1 was discovered to be the key upstream activator of the AMPK, thus, identifying LKB1 as a regulator of cell metabolism (Hawley *et al*, 2003; Hong *et al*, 2003; Woods *et al*, 2003; Shaw *et al*, 2004b). Adenosine monophosphate-activated protein kinase functions as a central metabolic regulator found in all eukaryotes that governs glucose and lipid metabolism and autophagy in response to alterations in nutrients and intracellular energy levels. Adenosine monophosphate-activated protein kinase is the only LKB1 substrate that is activated under low ATP conditions following nutrient deprivation or hypoxia and functions as a cellular rheostat maintaining energy homeostasis. Adenosine monophosphate-activated protein kinase is activated as well by CaMKK*β* in response to calcium flux (Hawley *et al*, 2005; Hurley *et al*, 2005; Woods *et al*, 2005). Adenosine monophosphate-activated protein kinase exists as a heterotrimeric complex composed of a catalytic subunit (AMPK*α*1, *α*2) and two regulatory subunits (AMPK*β*1, *β*2 and AMPK*γ*1, *γ*2, *γ*3). Adenosine monophosphate-activated protein kinase is activated upon the direct binding of ADP or AMP to a *γ* subunit where AMPK undergoes a conformational change leading to the phosphorylation of Thr172 on the activation loop of the *α* subunits (Hardie *et al*, 2011; Oakhill *et al*, 2011; Xiao *et al*, 2011). Upon cellular energy stress, AMPK activates catabolic pathways with the concomitant inhibition of anabolic metabolism, which serve to restore energy homeostasis (Mihaylova and Shaw, 2011; Hardie *et al*, 2012).

**Figure 2:**
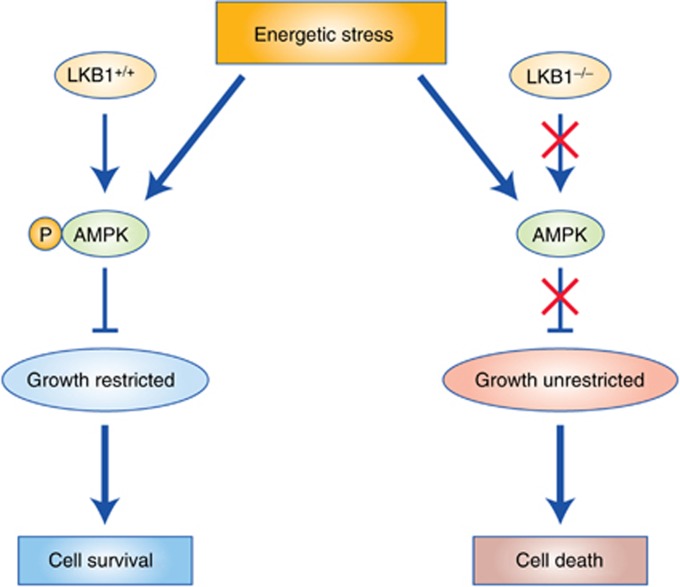
**LKB1-deficient cells show select sensitivity to energetic stress.** In the presence of energetic stress, LKB1-competent cells activate AMPK, which results in growth restriction and cell survival. LKB1-deficient cells in the presence of energetic stress fail to activate AMPK and thus continue with unrestricted growth leading to metabolic catastrophe and cell death.

Tumour cells lacking LKB1 are hypersensitive to apoptosis in culture following treatment with energy stress-inducing agents, presumably originating from an inability to restore ATP levels owing to AMPK deficiency (Shaw *et al*, 2004b). Thus, selective killing of LKB1-deficient tumour cells can be achieved by mimicking energy stress with small molecule AMPK agonists such as the AMP mimetic AICAR or the biguanide metformin and phenformin, which are both inhibitors of mitochondrial complex I (Dykens *et al*, 2008) (Table 1). LKB1 loss selectively sensitises human cell lines and genetically engineered mouse models (GEMMs) of NSCLC as well as mouse MC38 colon cancer and Lewis lung carcinoma cell lines by inducing energetic stress and metabolic catastrophe that resulted in cellular apoptosis (Algire *et al*, 2011; Shackelford *et al*, 2013b) (Figure 2). Adenosine monophosphate-activated protein kinase was recently shown to be degraded by the cancer-specific MAGE-A3/6-TRIM28 ubiquitinase in cancer cells leading to its downregulation and hypersensitivity to AMPK agonists AICAR and metformin (Pineda *et al*, 2015). Screening for reduced AMPK protein expression may be a clinically viable means to predict metabolic hypersensitivity in tumours. Metformin, (known as Glucophage clinically) is the most widely used type 2 diabetes drug in the world and is taken daily by approximately 120 million patients worldwide. Several retrospective studies revealed a strong correlation between reduced cancer risk and mortality in diabetic patients taking metformin (Evans *et al*, 2005; Bowker *et al*, 2006; Pollak, 2010; Currie *et al*, 2012), agreeing with early studies showing that biguanides suppressed naturally arising tumours in both transgenic and carcinogen-treated rodent cancer models (Schneider *et al*, 2001; Anisimov *et al*, 2005).

In order to inhibit complex I of ETC, metformin but not phenformin requires the cell membrane-bound organic cation transporter 1 (OCT1) for intracellular transport (Gorboulev *et al*, 1997; Segal *et al*, 2011). OCT1 is mainly expressed in the liver, while expression levels in human cancers are variable (Hayer-Zillgen *et al*, 2002). Phenformin, which is the more potent of the two biguanides, was banned in the United States in the late 1970s by the FDA owing to the rare but fatal lactic acidosis (Crofford, 1995; Owen *et al*, 2000). Phenformin-induced lactic acidosis occurs in 0.002% of patients in which 50% of those cases were fatal (Fimognari *et al*, 2006). Importantly, when compared with toxicities associated with front-line chemotherapies, and targeted chemotherapies such as EGRF tyrosine kinase inhibitors, where incidence of fatal adverse events is reported at 2% for patients receiving chemotherapy or EGFR tyrosine kinase inhibitor therapy, risk of phenformin-induced lactic acidosis might be outweighed by potential benefits offered by the drug (Qi *et al*, 2013). Of interest, toxicity to biguanides is associated with poor renal function and clearance of the drugs; however, this is routinely screened for in cancer patients as those with compromised renal function are excluded from severely toxic chemotherapies (Janus *et al*, 2010; Lalau, 2010; Ohara *et al*, 2012). The toxicity profiles of biguanides and potentially other metabolic therapies must be evaluated in the correct context if these drugs are to be repurposed as anti-cancer therapies. In addition to identifying the correct dosing of biguanides in cancer, it will also need to be determined in what clinical context metabolic therapies will be utilised – be it cancer prevention, neoadjuvant or adjuvant therapy, or treatment of late stage non-resectable tumours. The success of metabolic therapies in the clinic will likely be predicated on rigorous preclinical studies in rodent models of cancer that examine physiologically achievable concentrations of these drugs and identify their appropriate clinical application in oncology (Dowling *et al*, 2012).

### Deregulation of ULK1-mediated autophagy and mitophagy

**Figure 3:**
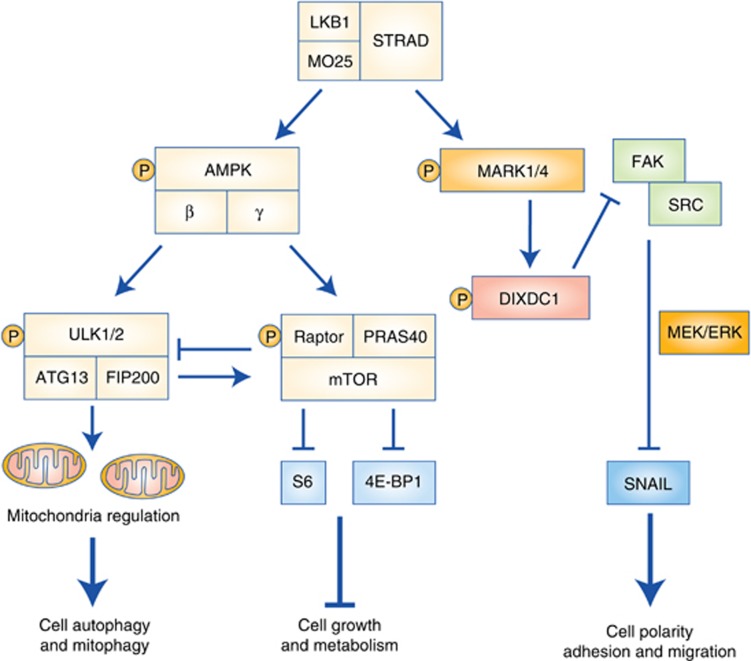
**LKB1 regulation of AMPK and MARK signalling pathways.** Phosphorylation of AMPK leads to phosphorylation of ULK1/2 and Raptor. As a result of phosphorylation of ULK1/2, autophagy and mitophagy are upregulated. Phosphorylation of Raptor by AMPK leads to downregulation of mTOR activity and its downstream targets S6 and 4E-BP1. LKB1 phosphorylates MARK1/4 leading to phosphorylation of DIXDC1 and inhibition of SNAIL protein through regulation of FAK and SRC kinases controlling both cell adhesion and migration.

Recent work in haematopoietic stem cells and NSCLC has shed light on the role of LKB1 as a regulator of mitochondrial homeostasis and autophagy. Deletion of *lkb1* in murine haematopoietic stem cells revealed mitochondrial defects including increased mitochondrial content and reduced mitochondrial membrane function (Gan *et al*, 2010; Gurumurthy *et al*, 2010; Nakada *et al*, 2010). Of interest, these studies implicate LKB1 as a regulator of stem cells. However, more studies are needed to understand how LKB1 regulates stem cells and how LKB1 inactivation deregulates cancer-initiating cells. It was then discovered that AMPK directly phosphorylates the Unc51-like kinases 1 and 2 (ULK1/2), key early regulators of autophagy and mitophagy (Egan *et al*, 2011) (Figure 3). Ampk^−/−^ and Ulk1^−/−^ murine embryonic fibroblasts displayed defects in autophagy and displayed the accumulation of aberrant mitochondria. Subsequent analysis of LKB1^−/−^ NSCLC tumour lines revealed inactivation of the AMPK-ULK1 signalling thus phenocopying the mitochondrial defects and reduced autophagy observed in Ampk^−/−^ and Ulk1^−/−^ murine embryonic fibroblasts (Shackelford *et al*, 2013b). These studies suggest that LKB1 inactivation and the resultant loss of AMPK-ULK1 signalling may lead to aberrant mitochondrial pools and decreased autophagy may compromise the ability of these tumours to supply cellular energetic demands through the TCA cycle. Autophagy was shown to be a critical source of nutrients to supply RAS-driven human tumours and Braf^V600E^-driven GEMMs of NSCLC (Guo *et al*, 2011; Strohecker *et al*, 2013). Using a synthetic lethal RNAi screening approach, White and colleagues identified KRAS and LKB1 co-mutant NSCLC tumours to be addicted to coatomer complex I (COPI)-dependent lysosome acidification that supplied macromolecules to the TCA cycle. KRAS/LKB1 mutant lung tumours were reliant on lysosomal degradation of macromolecules to fuel catabolic metabolism and showed sensitivity to inhibition of lysosomal maturation (Kim *et al*, 2013). Pharmacological activation of ER stress in Kras/Lkb1 mutant tumours resulted in unfolded protein response-mediated tumour cell death (Inge *et al*, 2014). These studies suggest that inhibiting autophagy, lysosomal acidification and inducing ER stress are vulnerabilities that may be effectively targeted in LKB1 mutant tumours.

## Targeting mTORC1 Hyperactivation in LKB1 Mutant Tumours

### Allosteric mTORC1 inhibitors

LKB1 negatively regulates mTORC1 kinase activity through AMPK phosphorylation of TSC2 and Raptor, therefore, LKB1 inactivation results in mTORC1 hyperactivation (Inoki *et al*, 2003; Corradetti *et al*, 2004; Shaw *et al*, 2004a; Gwinn *et al*, 2008) (Figure 3). As a result, the inhibition of mTOR has been extensively tested as a therapeutic approach to target LKB1^−/−^ tumour cells and mouse models of cancer. Preclinical studies examining rapamycin in Lkb1-deficient mouse models of cancer have yielded mixed results. Spontaneously arising hamartomas in Lkb1^+/−^ mice responded well (Wei *et al*, 2008; Shackelford *et al*, 2009b). Translation of these studies to treat hamartomas clinically with rapalogs has proven successful in tuberous sclerosis and lymphangioleiomyomatosis patients with the MILES trial (Bissler *et al*, 2008; McCormack *et al*, 2011), whereas early phase clinical trial for PJS patients was terminated owing to low enrolment. Rapamycin as a single agent has been shown to potently inhibit the growth and viability of endometrial carcinomas, oviductal neoplasias and papillary bladder tumours in Lkb1^−/−^ and Lkb1^−/−^;Pten^−/−^ GEMMs (Contreras *et al*, 2010; Shorning *et al*, 2011; Tanwar *et al*, 2012), as well as reduce the number and confluence of melanocytic lesions in BrafV600E; LKB1^−/−^ mice (Damsky *et al*, 2015), however, rapamycin failed to induce a therapeutic response in lung tumours from Kras^G12D^-driven LKB1-deficient GEMMs (Liang *et al*, 2010). Allosteric mTORC1 inhibitors have generally performed poorly in clinical trials as tumours do not sustain a durable response and become resistant likely owing to mTORC2-AKT-mediated reactivation of mTORC1 (Wander *et al*, 2011). For patients with LKB1 mutant tumours receiving rapalogs, it will be important to define the appropriate genetic and molecular landscape that dictates either response or resistance.

### mTOR catalytic kinase inhibitors

The mTOR catalytic kinase inhibitors that target both mTORC1 and mTORC2 or dual mTOR/PI3K inhibitors are predicted to show greater clinical efficacy than rapalogs for the treatment of LKB1 mutant tumours (Apsel *et al*, 2008; Maira *et al*, 2008; Feldman *et al*, 2009). Treatment of Kras-driven, PI3KCA mutant murine lung tumours with the dual PI3K/mTOR inhibitor BEZ235 resulted in the inhibition of both PI3K and mTOR signalling and regression of primary lung tumours (Engelman *et al*, 2008). However, for the Kras; Lkb1^−/−^ mouse lung tumour model, the PI3K/mTOR inhibitor BEZ235 was effective only when combined with the MEK inhibitor AZD2644 and dasatinib, inhibitor of Src-family of kinases (Carretero *et al*, 2010). Although LKB1 mutations are infrequent in breast cancer, PJS patients show an increased risk for the development of breast cancer (Sanchez-Cespedes, 2007). The mTOR catalytic kinase inhibitors AZD8055 successfully inhibited tumour growth in Lkb1^−/−^ NIC mouse breast tumour model (Andrade-Vieira *et al*, 2014). Of interest, both rapamycin and the dual PI3K/mTOR inhibitor BEZ235 inhibited tumour growth in an Lkb1/Pten-deficient GEMM of endometrial cancer agreeing with a previous study that showed that single-therapy rapamycin effectively reduced tumour burden in Lkb1-deficient GEMM of endometrial ADC (Contreras *et al*, 2010; Cheng *et al*, 2014). Lastly, treatment of the Kras/Lkb1 GEMMs with the multi-target receptor tyrosine kinase inhibitor sunitinib (Sutent) was effective and reduced tumour size, increased tumour necrosis and slowed tumour progression in primary lesions demonstrating that receptor tyrosine kinases may be targeted in addition to the PI3K/mTOR pathway (Gandhi *et al*, 2009).

## LKB1 Substrates – The Potential of Old and new Therapeutic Targets

### Adenosine monophosphate-activated protein kinase-like kinases

While deregulation of the AMPK pathway has received considerable attention, recently studies investigating AMPK's other siblings have made significant progress towards mapping the LKB1 signalling pathway. There has been considerable work to elucidate the function of AMPK-like kinases, however, much of this work has been performed on an LKB1 wild-type background. An increasing number of studies suggest that the 12 additional AMPK-like kinases that are phosphorylated by LKB1 do have an important role in cancer development and progression. BRISK1 and BRISK2 have roles in neural development, but a recent report showed that decreased expression in samples from breast cancer patients negatively correlates with clinical outcomes (Wang *et al*, 2014). High levels of expression of NUAK1 (ARK5) have been reported to correlate with poor prognosis in patients with hepatocellular carcinoma (Liu *et al*, 2012a; Cui *et al*, 2013), glioma (Lu *et al*, 2013), colorectal cancer (Kusakai *et al*, 2004; Roh *et al*, 2010), multiple myeloma (Suzuki *et al*, 2005) and pancreatic cancer (Suzuki *et al*, 2004). Reports on the role of NUAK2 in tumours of different origin vary: in melanoma, NUAK2 (SNARK) levels correlate negatively with patient survival (Namiki *et al*, 2011), whereas in ovarian cancer patients, an opposite trend was reported (Emmanuel *et al*, 2011). SIK1, SIK2 (QIK) and SIK3 (QSK) have been reported to have a role in metastasis in gastric ADCs (Selvik *et al*, 2014) and mitotic progression in prostate and ovarian cancers (Ahmed *et al*, 2010; Chen *et al*, 2014; Bon *et al*, 2015). Additionally, SIK1 was shown to be required for P53-dependent anoikis, and RNAi-mediated gene silencing of SIK1 resulted in increased lung micrometastases in xenografts injected with transformed human mammary epithelial cells. Expression of conditionally active SIK1 in LKB1 mutant A549 cell lines suppressed invasion and pulmonary metastasis, thus implicating an important role for the SIK1 in progression and metastasis of LKB1-deficient tumours (Cheng *et al*, 2009). The maternal embryonic leucine-zipper kinase (MELK) expression negatively correlates with patient survival in cases of acute myeloid leukaemia (Alachkar *et al*, 2014), gastric cancer progression (Du *et al*, 2014), high grade prostate cancer (Kuner *et al*, 2013) and breast cancer (Pickard *et al*, 2009). Finally, SNRK levels have been reported to be higher in colon cancer lines compared with normal cells (Rines *et al*, 2012).

Recent studies point to deregulation of the group of AMPK-like kinases called MAP/microtubule affinity-regulating kinases 1–4 (MARKs1–4; also known as PAR1) following LKB1 inactivation as having an integral role in metastasis. The link between deregulation of MARKs and metastasis is important because the inactivation of Lkb1 promotes metastasis in the Kras^G12D^ mouse model of lung cancer; however, the exact mechanism(s) have not been uncovered (Ji *et al*, 2007). The MARKs are cellular regulators of polarity and are conserved from yeast to mammals (Martin-Belmonte and Perez-Moreno, 2012). A recent study investigating downstream substrates of the MARK kinase family, identified DIXDC1, a scaffold protein that localises to focal adhesions as a direct target of MARK1 and of MARK4 (Goodwin *et al*, 2014) (Figure 3). Inactivation of LKB1 resulted in an AMPK-independent increase in levels of Snail1 that were dependent on MARK1 and MARK4. The authors demonstrate that inactivation of the LKB1-MARK-DIXDC1 signalling axis resulted in the activation of FAK and SRC. Activation of FAK led to MEK/ERK-dependent upregulation of SNAIL resulting in increased cell migration and invasion *in vitro* and *in vivo* in lung colonisation assay. Low DIXDC1 expression significantly correlated with decreased NSCLC patient overall survival suggesting that the MARK kinase family and DIXDC1 are important clinical biomarkers in cancer. Additionally, it was shown that the loss of LKB1 results in FAK phosphorylation that can be repressed by re-expressing LKB1 or by treating cells with FAK inhibitor PF-573228 (Kline *et al*, 2013). This study links an earlier study by Carretero *et al* (2010) who demonstrated that LKB1 mutant NSCLC tumour cell lines are sensitive to RNAi-mediated silencing of FAK and SRC. Treatment of Kras^G12D^-driven, Lkb1^−/−^ (Kras/Lkb1) mouse models of lung cancer with the combination of the SRC inhibitor dasatinib, MEK inhibitor AZD6244, and the dual PIK3CA and mTOR inhibitor BEZ235 resulted in decreased primary and metastatic lung tumours (Carretero *et al*, 2010). Similar results were observed in a mouse melanoma model, where loss of LKB1 correlated with increased phosphorylation of SRC Family Kinase (SFK) Yes, which resulted in increased cell migration and invasion. Pan-SFK inhibitor dasatinib did not inhibit tumour metastasis *in vivo* owing to inadequate inhibition of Yes kinase (Liu *et al*, 2012a, 2012b). Lastly, a genetic screen identified that the LKB1/MARK signalling axis regulates the Hippo (MST1/2 in humans) Yap pathway. Activation of YAP was consistently seen in Lkb1-deficient human PJS polyps and tumours as well as in mouse models of NSCLC and pancreatic ductal ADC (Mohseni *et al*, 2014). In sum, these studies demonstrate MARK1 kinases as important regulators of metastatic potential.

## Preclinical Models for LKB1-Deficient Tumours

### Lkb1-deficient mouse models of cancer

Germ-line and conditional deletion of *lkb1* in mice have confirmed that LKB1 inactivation has an important role in tumourigenesis in a variety of tissues. Genetically engineered mouse models have proven to be a valuable tool to understand the molecular basis of disease following inactivation of LKB1. Biallelic loss of *lkb1* is embryonic lethal in mice, yet, heterozygous lkb^+/−^ mice develop gastrointestinal hamartomas that closely recapitulate the pathophysiology of the PJS patients (Ylikorkala *et al*, 2001; Bardeesy *et al*, 2002; Jishage *et al*, 2002; Miyoshi *et al*, 2002; Rossi *et al*, 2002; Katajisto *et al*, 2008). Conditional, biallelic deletion of *lkb1* in the lung tissue accelerated Kras^G12D^-driven lung tumours in mice and led to heterogeneous tumour development of ADC, squamous and large cell carcinomas that closely mirror the human disease (Ji *et al*, 2007). Conditional inactivation of *lkb1* in a number of tissues in mice have generated tumours in breast, pancreas, prostate, skin and bone. (For reviews, see (Ollila and Makela, 2011; Shackelford, 2013). The accuracy of these mouse models to human disease is an important contributing element to the advances the field of LKB1 research has made in the last decade and a half.

### Patient-derived xenografts and organoid models

In addition to GEMMs, integrating patient-derived xenografts (PDXs) models will provide comprehensive *in vivo* models to study LKB1 inactivation in primary human tumours. Patient-derived xenograft models take with great efficiency in cancer with frequent LKB1 mutations such as NSCLC, pancreas and colon (Fu *et al*, 1991, 1992) (Gandara *et al*, 2015) and patient-derived cell lines are amenable to 3D culture (Zhang *et al*, 2012). Identification of LKB1 inactivation in PDX models will require deep sequencing and/or functional studies. Additionally, organoid models in colon and pancreatic ADC have been established, and it is likely these models could be used to successfully bridge *in vitro* and *in vivo* studies of Li *et al* (2014) and Boj *et al* (2015).

### The advent of the co-clinical trial

The use of GEMMs in co-clinical studies may provide an efficient platform to begin evaluating single and combination therapies to which Lkb1-deficient tumours are both responsive and resistant. Seminal studies published in recent years have effectively integrated the use of GEMMs to mirror human clinical trials for NSCLC and acute promyelocytic leukaemia (Nardella *et al*, 2011). In a seminal study by Chen *et al* (2012), Kras^G12D^-driven GEMMs were used in a co-clinical trial testing the combination of docetaxol+selumetinib in patients identified as having lung tumours positive for KRAS mutations. The authors stratified patients by KRAS single mutation and KRAS/LKB1 or KRAS/P53 co-mutations and mirrored human therapies using Kras^G12D^ (K), Kras^G12D^/Lkb1^−/−^ (KL) and Kras^G12D^/p53^−/−^ (KP) GEMMs. Surprisingly, the authors discovered that K and KP lung tumours were responsive to docetaxol+selumetinib therapy, whereas KL tumours were unresponsive to therapy. Detailed molecular analysis revealed low activation of the MEK/ERK pathway in KL lung nodules, thus explaining the lack of therapeutic response. Interestingly, in a separate pre-clinical study comparing the response of K, KL and KP GEMMs to phenformin, it was discovered that KL tumours showed a significant response to phenformin, while K and KP lung tumours remained resistant, thus demonstrating that Lkb1 inactivation determined sensitivity to phenformin (Shackelford *et al*, 2013a). These studies underscore the importance of stratifying lung tumours by genetic mutations and incorporating GEMMs as an integral part of co-clinical trials.

## Looking Forward – Identifying new Pathways to Target

Continuing our understanding on how LKB1 inactivation deregulates basic cellular functions such as metabolism and growth will be an important next step. A synthetic lethal screen performed on Kras/p53/Lkb1 mutant GEMM derived NSCLC tumour lines and identified Deoxythymidylate kinase as a top hit thus coupling LKB1 inactivation to dependency on folate metabolism in order to supply nucleotides required for cellular replication (Liu *et al*, 2013). The extracellular matrix remodelling protein lysyl oxidase was found to be highly elevated in Kras/Lkb1 GEMMs and inhibition of lysyl oxidase with BAPN also impaired tumour progression (Gao *et al*, 2010). Future studies are needed to comprehensively profile the intracellular tumour environment such as metabolism as well as the extracellular microenvironment and such studies will likely shed light on new tumour vulnerabilities that occur following LKB1 loss.

As with any therapy, we expect that some cells within tumours will develop resistance to LKB1-targeted therapy. Specific resistance mechanism would depend on the therapy used. Because the majority of therapies targeting LKB1 vulnerabilities concentrate on disrupting metabolism at some cellular level, one could predict that metabolic adaptation will be the likely course of resistance in LKB1^−/−^ tumours. While resistance to targeted therapies such as mTOR inhibitors are likely to follow canonical pathways such as activation of AKT or mTORC2, metabolic-based therapies will induce metabolic adaptation (O'Reilly *et al*, 2006; Rodrik-Outmezguine *et al*, 2011). Therefore, it will be necessary to map the metabolic dependencies in LKB1 mutant tumours in order to identify and predict metabolic resistance. For example, resistance to biguanides that target complex I of ETC induce a metabolic increase in glycolytic metabolism (Dykens *et al*, 2008; Shackelford *et al*, 2013a). *In vitro* cell-based screens using 2D and 3D cell culture and possibly organoids could be set up with cells exposed to targeted therapies for prolonged time allowing for resistance to develop. Coupling gene expression to analysis of signal transduction and metabolic pathways would provide a rapid and robust means to identify novel and canonical resistance mechanisms that could be tested *in vivo*.

Combining therapy targeting LKB1 tumours with currently available chemotherapy is an area that is unexplored and needs to be addressed by the field. To date, no study has been performed to identify specific chemotherapies that selectively target LKB1^−/−^ tumours, however, large-scale analyses such as the cancer cell line encyclopaedia represent studies that may offer clues (Barretina *et al*, 2012). Identification of novel strategies that combine chemotherapy with targeted or metabolic therapies that are both effective and carry low toxicities may be clinically beneficial.

The anti-PD-1 agent nivolumab was recently FDA-approved for the treatment of squamous cell lung cancer. Immunotherapy therapies targeting PD1 and PDL1 in NSCLC have shown great promise in the clinic (Topalian *et al*, 2012; Xu *et al*, 2014). Anti-PD-1 and PD-L1 therapies may prove effective against LKB1 mutant squamous lung tumours as a recent study describing an Lkb1/Pten-deficient GEMM of squamous cell lung cancer found that tumours expressed high PD-L1 levels (Xu *et al*, 2014). Combination therapies adding together different therapeutic approaches such as targeted therapies, metabolic therapies and immunotherapies are likely to yield the best clinical results and may be tested pre-clinically using Lkb1-deficient GEMMs.

An additional step of stratification and treatment of LKB1-deficient tumours may be to group them with co-mutations that frequently arise in genes such as KRAS and BRG1 (Carretero *et al*, 2004; Medina *et al*, 2008). A recent study described an allosteric irreversible small molecule inhibitor, compound 12, that selectively binds to the cysteine of oncogenic KRAS^G12C^ resulting in preferential growth inhibition and apoptosis of KRAS^G12C^ mutant tumour lines (Ostrem *et al*, 2013). Functional epigenetic studies in lung ADC identified BRM/SMARCA2 and EZH2 potential targets in BRG1 mutant lung tumours (Hoffman *et al*, 2014; Fillmore *et al*, 2015). Future studies examining the combination of novel inhibitors of KRAS or of epigenetic regulators in combination with energy stress agents and/or targeted therapies may prove an effective strategy for targeting tumours for LKB1/KRAS^G12C^ or LKB1/BRG1 co-mutated tumours.

## Concluding Remarks

Work from preclinical studies in cell lines, xenografts and GEMMs predict that metabolic therapies, targeted therapeutics targeting LKB1 vulnerabilities will be most effective in combination. Immunotherapies have gained considerable traction as highly effective therapies and were recently approved for the treatment of melanoma and NSCLC. Achieving personalised treatments for patients harbouring LKB1 mutations will require defining vulnerabilities, identifying effective drugs that target these vulnerabilities and identifying appropriate research platform(s) to test novel therapeutics *in vivo*.

As every single approach has its limitations, it is reasonable to assume that systematic and efficient translation of new therapies targeting LKB1 vulnerabilities will require the development and integration of both cell-based and mouse-based research platforms. The use of 2D and 3D culture systems as well as the development of organoid models hold promise for accurately bridging *in vitro* and *in vivo* studies. Novel combination therapies targeting LKB1 vulnerabilities in cancer will need to be rigorously tested in both cell-culture-based and animal models. Because most tumours eventually develop resistance to therapy, it will be important to choose pre-clinical *in vivo* models that readily develop resistance and accurately model the heterogeneity present in most tumours. Although xenografts remain a highly effective *in vivo* means to screen isogenic tumour lines, they should be used in combination with autochthonous models such as GEMMs or with PDXs. This will likely facilitate defined treatment modalities that selectively target this large subset of tumours. Finally, using imaging-based preclinical studies in animal models, such as GEMMs and PDXs, to rigorously test new therapeutic strategies hold great promise for efficiently translating novel drugs that target LKB1 vulnerabilities into clinical trials (de Jong *et al*, 2014).

## Figures and Tables

**Figure 1:**
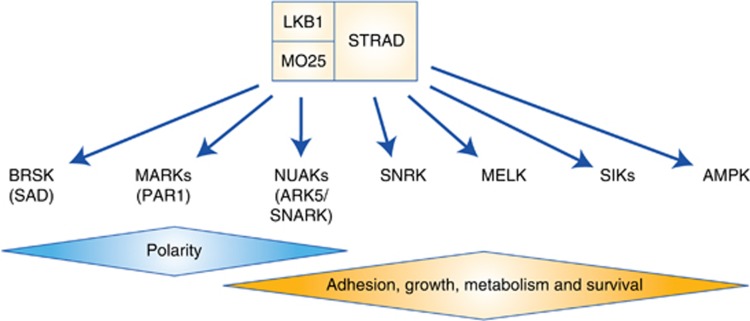
**LKB1 regulates an AMPK-like family of kinase.** LKB1 in complex with STRAD and MO25 phosphorylates AMPK and AMPK-like kinases to regulate polarity, adhesion, growth, metabolism and cell survival.

**Table 1:** Drugs that can be used to target LKB1-deficient tumours

**Drug(s)**	**Target(s)**	**Vulnerability**	**Reference**
Metformin, Phenformin	Complex I of ETC	Energetic stress	Shaw *et al*, 2004b; Algire *et al*, 2011; Shackelford *et al*, 2013a
Bafilomycin A, saliphenylhalamide A	Lysosomal acidification	Compromised autophagy, mitochondrial metabolism	Guo *et al*, 2011; Kim *et al*, 2013
Rapamycin	mTORC1	Dependence on high mTOR kinase activity	Wei *et al*, 2008; Shackelford *et al*, 2009a; Contreras *et al*, 2010; Liang *et al*, 2010; Shorning *et al*, 2011; Tanwar *et al*, 2012; Damsky *et al*, 2015
AZD8055 BEZ235	mTOR catalytic kinase, dual PI3K and mTOR kinase	Dependence on high mTOR kinase activity	Carretero *et al*, 2010; Andrade-Vieira *et al*, 2014; Cheng *et al*, 2014
AZD6224/BEZ235/Dasatinib	MEK, PI3K/mTOR and SRC	Combinatorial targeting of signalling pathways	Carretero *et al*, 2010; Chen *et al*, 2012; Liu *et al*, 2012b
Sunitib	Multi-target receptor tyrosine kinases	Inhibition of angiogenesis	Gandhi *et al*, 2009
Tunicamycin, brefeldin A, 2-deoxyglucose	ER, glycolysis, unfolded protein response	ER stress activation, metabolic stress	Inge *et al*, 2014
BAPN	Lysyl oxidase	ECM	Gao *et al*, 2010

Abbreviations: ECM=extracellular matrix; ER=endoplasmic reticulum; ETC=electron transport chain. Drugs along with their known targets are grouped based on LKB1-dependent vulnerability that is exploited by each class of drugs.
